# Description of Pre-Adult Stages of the Coconut Bug, *Pseudotheraptus wayi*

**DOI:** 10.1673/031.013.9101

**Published:** 2013-09-23

**Authors:** James Peter Egonyu, Jacques Kabaru, Lucy lrungu, Fabian Haas

**Affiliations:** 1 ICIPE-African Insect Science for Food and Health, P.O. Box 30772, 00100 Nairobi, Kenya; 2 School of Biological Sciences, University of Nairobi, P.O. Box 30197, 00100 Nairobi, Kenya

**Keywords:** adult, development, egg, identification, instars

## Abstract

The coconut bug, *Pseudotheraptus wayi* Brown (Hemiptera: Heteroptera: Coreidae), is a serious pest of a number of crops in Eastern and Southern Africa. Both adults and nymphal stages are destructive because they suck sap from their hosts. The identity of the pest is currently based exclusively on the description of adults. This paper describes eggs and instars of *P. wayi*, with the goal to enhance identification of all stages for effective monitoring and management of the pest. Morphological illustrations are presented, and differences among the instars, as well as their relationship with the adult stage, are discussed.

## Introduction

The coconut bug, *Pseudotheraptus wayi* Brown (Hemiptera: Heteroptera: Coreidae), is a polyphagous pest of cashew, *Anacardium occidentale*, coconut, *Cocos nucifera*, macadamia, *Macadamia integrifolia*, carambola, *Averrhoa carambola*, pecan, *Carya illinoinensis*, cinnamon, *Cinnamomum verum*, loquat, *Eriobotrya japonica*, mango, *Mangifera indica*, avocado, *Persea americana*, guava, *Psidium guajava*, cocoa, *Theobroma cacao*, and various wild legumes (Way 1953; Hill 1983, 2008; Van der Meulen 1992; Van der Meulen and Schoeman 1994; CABI 2005; Nyambo 2009). Both nymphs and adults of *P. wayi* suck sap from young stems, leaves, inflorescences, and fruits, and inject toxins into the plant tissues, causing wilting and necrosis (Hill 1983, 2008; Way 1953). The damage caused by this species can be very significant depending on the host. It can contribute to 80% nut yield loss on cashew (Nyambo 2009), 76.2% damage on ripe avocado (Van der Meulen and Schoeman 1994), 52.4% damage on guava fruits (Van der Meulen 1992), and 99.8% coconut nut fruit abortion (Way 1953).

The geographical distribution of *P. wayi* is Eastern and Southern Africa (CABI 2005), thereby making it a quarantine pest in most countries. Thorough identification of this pest is vital for its management and prevention of it spreading. Surprisingly, only the adult morphology of *P. wayi* has been described (Brown 1955), even though each of the 5 instars is equally destructive (Way 1953; Hill 1983, 2008). This paper therefore presents morphological descriptions of pre-adult instars in order to provide information necessary for the identification of *P. wayi* at all life stages. For completeness, images of adults are presented, and features that relate to instars are described. However, readers are referred to Way (1953) for detailed adult morphology.

## Materials and Methods

Specimens of *P. wayi* were obtained from the insectary at the International Centre of Insect Physiology and Ecology from a colony that was initiated using insects collected from cashew nurseries and orchards at the Kenya Agricultural Research Institute, Mtwapa Research Centre. Mtwapa Research Centre is situated 20 km north of Mombasa City in Kilifi district at 3° 55′ S; 39° 44′ E; 15 m a.s.l. (KARI Mtwapa 2004).

The studies were conducted at the Biosystematics Support Unit laboratory of the International Centre of Insect Physiology and Ecology. Five specimens of each instar preserved in 80% ethanol and 5 live eggs were photographed and measured using a Leica® Microsystems EZ4D Microscope connected to a computer using the recommended Leica® Application Suite Software, version 1.5 (Leica Microsystems, www.leicamicrosystems.com). Egg length and width were measured from the two tips and the widest point, respectively. Nymphal and adult pronotal length and width were measured from the medial longitudinal axis and the widest posterior end, respectively.

Synthlipsis (shortest inter ocular distance) was also measured. For antennae, both left and right antennomeres were measured. The wet weights of eggs, nymphs, and adults were determined using 10 individual live specimens. Voucher specimens of nymphs and adults were deposited in the Biosystematics Support Unit collection of the International Centre of Insect Physiology and Ecology (Catalogue No.: Egonyu et al. 2011). Live specimens were photographed.

## Results and Discussion

### **Egg** (Figure 1A)

Oval, smooth, cream, but turning reddishbrown prior to hatching. Egg length was previously estimated at 1.5 mm (De Villiers 1992, cited by CABI 2005), but literature on weight and width is not available.

### **First instar** (Figure 1B)

Body lanceolate, generally reddish-brown with head-thorax region darker and abdominal terga with whitish patches. Ratio of length of head:thorax:abdomen approximately 1:1:2. Reddish-brown punctation dorsally on the head (Table 1). Tylus porrect, protruding well forward between the antennal tubercles as in adults (Brown 1955). Antennae noncylindrical but rather flattened laterally, as opposed to those of adults. Antennomeres uniformly reddish-brown, with second clavate and third conspicuously widest. Eyes and ocelli reddish-pink as in adults (Brown 1955). Rostrum 4-segmented, basal segment thickest and with reddish-brown punctation, second and third sub-equal in length and both shorter than basal segment, fourth longest with a dark brown tip. About one-fifth of rostrum extending posteriorly beyond metacoxae when at rest. An orange Y-shaped ecdysial line is present dorsally through head and thorax.

**Table 1.**
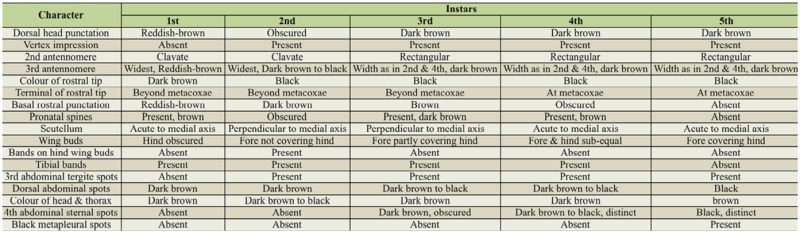
Key qualitative features of *Psudotheraptus wayi* instars.

Pronotum broader than long, its anterior and posterior width sub-equal, and medial length sub-equal to that of mesonotum. A pair of prominent brown spines on either side of pronotum. Mesonotum wider posteriorly, with minor constrictions between scutellum and forewing buds, the former extending farther posteriorly than the latter and acute to medial axis. Hind wing buds obscured. Metanotum less distinct than pro- and mesonota but also wider posteriorly, and conjoining first abdominal sclerite with a gentle constriction. Fore-, mid-, and hind legs approximately same length. Ratio of length of femora:tibiae:tarsi approximately 4:3:1 in forelegs and 2:1:1 in mid- and hind legs. Femora and tarsi brown, tibia with reddish-brown and white bands.

Ten abdominal terga, with 1 to 3 not easily discernible and 4 or 5 the widest; margin serrated. Posterior half of abdomen with more whitish patches at center than the rest. A pair of dome-like scent glands with posteriorly curving sutures present at mid posterior ends of fourth and fifth abdominal terga, about equal in size, each with a brown spot on either side. Coreid nymphs normally possess dorsal abdominal scent glands opening between tergites 4/5 and 5/6 (Cobben 1978, cited by Aldrich 1988). A pair of sub-equal dark brown spots laterally on either sides of fifth abdominal tergite.

### **Second instar** (Figure 1C)

Body oblanceolate, generally brown with head-thorax region and third antennomere dark brown to black. Antennal shape and third antennomere as in 1st instar, third antennomere conspicuously widest. Ocelli, dorsal head punctation, and pronotal spines obscured. A well-marked median longitudinal impression on vertex between eyes as in adults (Brown 1955). Rostral tip black, and punctation on basal segment dark brown, ecdysial line reddish-brown.

Scutellar-wing bud constrictions sharper than in 1st instar, forewing buds and scutellum sub-equal in length posteriorly. Posterior end of scutellum approximately perpendicular to medial axis. Metanotum distinct, about half the length of pro- and mesonota, with reddishbrown longitudinal bands at hind wing buds, and conjoining first abdominal sclerite with a sharper constriction than in 1st instar. Hind wing buds not covered by forewing buds. Fore- and mid-legs approximately same length, but shorter than hind legs. All legs with same ratio of length of femora:tibiae:tarsi, which is approximately 2:2:1. Femora pinkish-brown with reddish-brown punctation, tibia as in 1st instar, tarsi reddish-brown.

Abdominal terga 1 to 3 more distinct than in 1st instar; an additional brown spot laterally on either sides of third abdominal tergite and closer to the medial axis than their counterparts on fifth abdominal tergite; all 6 spots as well as those on dorsal abdominal scent glands darker brown than in 1st instar. Abdominal margins as in 1 st instar.

### **Third instar** (Figure 1D)

Body ovate, generally pinkish-brown with head-thorax region and third antennomere lighter than in 2nd instar. Dorsal head punctation dark brown. Tylus as in 1st and 2nd instars. Eyes and ocelli as in 1st instar. Antennal shape as in 1st and 2nd instars, basal antennomere widest, and the rest are subequal in width. Ecdysial line and dorsal head impression as in 2nd instar. Basal rostral punctation smaller and lighter than in 2nd instar, but rostral tip black as in 2nd instar.

Pronotal spines dark brown. All thoracic terga wider posteriorly. Constriction between scutellum and forewing buds more distinct than in 1st and 2nd instars with forewing buds longer than scutellum posteriorly. Posterior end of scutellum as in 2nd instar. Length of fore-, mid-, and hind legs as in 2nd instar. Ratio of length of femora:tibiae:tarsi approximately 3:2:1 in fore- and mid-legs, and 4:2:1 in hind legs. Patterns and color of legs as in 2nd instar.

Dorsal abdominal spots on terga and scent glands darker than in 1st and 2nd instars. Pair of dark brown spots barely visible on fourth abdominal sternum.

### **Fourth instar** (Figure 1E)

Body shape and color as in 3rd instar, with head-thorax region and third antennomere lighter than in 2nd and 3rd instars. Ratio of length of head:thorax:abdomen approximately 1:1:3. Antennal shape as in 1st-3rd instar, basal and second antennomeres light brown, third dark brown, distal with reddish-brown tint, and width as in 3rd instar. Dorsal head punctation as in 3rd instar, tylus as in 1st-3rd instars. Eyes and ocelli as in 1st and 3rd instars. Dorsal head impression and ecdysial line as in 2nd and 3rd instars. Rostrum terminating at metacoxal area, punctation on basal segment sparsely visible, and tip black as in 2nd and 3rd instars.

Pronotal spines lighter than in 2nd and 3rd instars. Fore- and hind wing buds sub-equal and reaching first abdominal tergum. Posterior terminal of scutellum acute to medial axis as in 1st instar. Length of fore-, mid- and hind legs as in 2nd and 3rd instars. Ratio of length of femora:tibiae:tarsi in fore- and mid-legs as in 3rd instar and approximately 3:3:1 in hind legs. Patterns on legs as in 2nd and 3rd instars, but femora and tibial white bands turn light pink.

More smaller dorsal abdominal spots present in some specimens, these and other dorsal abdominal and scent gland spots darker than in 1st-3rd instars. Spots on fourth abdominal sternum distinctly visible and dark brown to black.

### **Fifth instar** (Figure 1F)

Body elliptical, with color of head-thorax region and third antennomere not as distinct from the rest as in 1st-4th instars. Ratio of length of head:thorax:abdomen approximately 1:2:3. Antennal shape and tylus as in 1st-4th instars, width of antennomeres and dorsal head punctation as in 3rd and 4th instars. Eyes and ocelli as in 1st, 3rd, and 4th instars. Dorsal head impression as in 2nd-4th instars. Rostrum as in 4th instar but without punctation at basal segment. Ecdysial line whitish and less pronounced than previous instars. Pronotal anterior width shorter than its medial length as well as that of mesonotum. Metanotal medial length less than a quarter those of meso- and pronota. Pronotal spines absent. Scutellar terminal acute to the medial axis as in 1st and 4th instar. Forewing buds reaching second or third abdominal terga and longer than hind ones. Length of fore-, mid-, and hind legs as in 2nd to 4th instars. Ratio of length of femora:tibiae:tarsi approximately 4:2:1 in forelegs, 4:4:1 in mid-legs, and same as 4th instar in hind legs. All legs lack femoral punctation and tibial bands, femora light pink and darker distally, tibiae and tarsi brown. Dorsal abdominal spots as in 4th instar and are black.

Ventral sclerites more distinct and smoother than in previous instars, and are light pink with a pair of black spots on metapleura and fourth abdominal sternum. The metapleural spots are generic diagnostic features of adults (Brown 1955).

### **Adults** (Figures 1 G, H)

The lengths of antennomeres agree with Brown (1955) in that the third segment is distinctly shorter than the others. Distal antennomere the most hairy. The spots on fourth abdominal sternum found on 3rd-4th instars, and dorsal abdominal spots absent, dorsal scent glands not easily discernible. In most coreids, dorsal abdominal scent glands, though present, often do not function in adults, and the metathoracic scent glands opening laterally on metapleura become functional (Staddon 1979; Aldrich 1988).

Length of fore-, mid-, and hind legs as in 2nd5th instars. Ratio of length of femora:tibiae:tarsi in females approximately 1:1:1 in fore- and mid-legs, and 2:2:1 in hind legs, while that in males is approximately 2:2:1 in all legs. Color of legs as in 5th instar. Adult legs were previously not described.

### Conclusion

Pre-adult stages of *P. wayi* have a number of distinct morphological features, as opposed to the previous assertion that its nymphs are morphologically very similar (De Villiers 1992, cited by CABI 2005). The distinct features of each stage of *P. wayi* can be of great help in effective identification of the pest for proper monitoring and management. Similar studies on other species of the genus would be of value in developing a dichotomous key for all stages. Furthermore, the revelation of morphological transformations in the development stages may be a recipe for identification of juvenile hormones triggering the transformations, which could be used in managing the pest through growth regulation.

**Figure 1.**
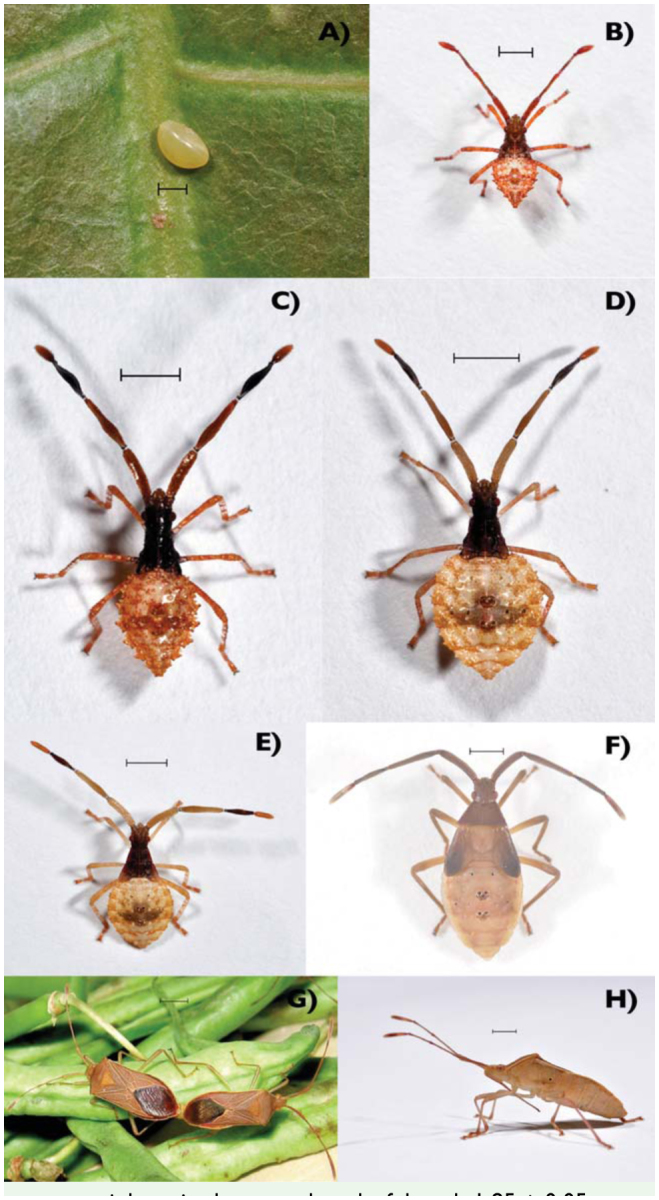
**A).** An egg of *Pseudotheraptus wayi* deposited on a cashew leaf; length 1.85 ± 0.05 mm, width 1.26 ± 0.02 mm, weight 1.27 ± 0.07 mg; Photo: Fabian Haas; scale bar: 1 mm. **B).** First instar of *P. wayi;* weight 1.09 ± 0.05 mg; antennomeres (from proximal to distal) 0.81 ± 0.03 mm, 0.95 ± 0.02 mm, 0.77 ± 0.01 mm, 0.6 ± 0.01 mm; synthlipsis 0.46 ± 0.01 mm; pronotum 0.25 ± 0 mm long, 0.88 ± 0.14 mm wide; Photo: Fabian Haas; scale bar: 2 mm. **C).** Second instar of P. *wayi;* weight 3.37 ± 0.64 mg; antennomeres 1.27 ± 0.02 mm, 1.59 ± 0.02 mm, 1.29 ± 0.02 mm, 0.74 ± 0.01 mm; synthlipsis 0.61 ± 0.01 mm; pronotum 0.38 ± .02 mm long, 0.85 ± 0.02 mm wide; Photo: Fabian Haas; scale bar: 1 mm. **D).** Third instar of *P. wayi;* weight 6.4 ± 0.22 mg; antennomeres 1.72 ± 0.02 mm, 2.1 I ± 0.02 mm, 1.52 ± 0.53 mm, 0.79 ± 0.09 mm; synthlipsis 0.68 ± 0 mm; pronotum 0.52 ± .01 mm long, 1. 14 ± 0.01 mm wide; Photo: Fabian Haas; scale bar: 1 mm. **E).** Fourth instar of *P. wayi;* weight 25.18 ± 2.783 mg; antennomeres 2.21 ± 0.042 mm, 2.67 ± 0.07 mm, 1.81 ± 0.04 mm, 1.41 ± 0.06 mm; synthlipsis 0.8 ± 0.01 mm; pronotum 0.79 ± 0.04 mm long, 1.81 ± 0.04 mm wide; Photo: Fabian Haas; scale bar: 2 mm. **F).** Fifth instar of *P. wayi;* weight 47.96 ± 3.52 mg; synthlipsis 0.92 ± 0.02 mm; antennomeres 2.82 ± 0.05 mm, 3.1 2 ± 0.08 mm, 2.12 ± 0.05 mm, 2.17 ± 0.051 mm; pronotum 1.25 ± 0.07 mm long, 3.06 ± 0.03 mm wide; Photo: Fabian Haas; scale bar: 2 mm. **G).** A couple of *P. wayi* (♀ on the left) mating on French bean pods used for feeding at the insectary; weight ♀: 54.68 ± 1.87 mg, ♂: 44.02 ± 1.78 mg; antennomeres ♀: 2.79 ± 0.07 mm, 3.21 ± 0.03 mm, 2.1 ± 0.04 mm, 2.32 ± 0.09 mm; ♂: 2.72 ± 0.1 mm, 3.27 ± 0.07 mm, 2.21 ± 0.03 mm, 3.04 ± 0.06 mm; synthlipsis ♀: 1.08 ± 0.04 mm, *♂:* 1.04 ± 0.05 mm; length of pronotum ♀: 2.2 ± 0.12 mm, *♂:* 2.5 ± 0.06 mm; width of pronotum ♀: 4.19 ± 0.19 mm, *♂:* 4.26 ± 0.11 mm; Photo: JP Egonyu; scale bar: 2 mm. **H).** Side view of a ♀ P. *wayi;* Photo: Fabian Haas; scale bar: 2 mm. High quality figures are available online.
